# Immunomodulation of Autoimmune Arthritis by Herbal CAM

**DOI:** 10.1155/2011/986797

**Published:** 2010-12-22

**Authors:** Shivaprasad H. Venkatesha, Rajesh Rajaiah, Brian M. Berman, Kamal D. Moudgil

**Affiliations:** ^1^Department of Microbiology and Immunology, University of Maryland School of Medicine, HSF-1, Suite 380, 685 W. Baltimore st., Baltimore, MD 21201, USA; ^2^Center for Integrative Medicine, University of Maryland School of Medicine, East Hall, W. Lombard st., Baltimore, MD 21201, USA

## Abstract

Rheumatoid arthritis (RA) is a debilitating autoimmune disease of global prevalence. The disease is characterized by synovial inflammation leading to cartilage and bone damage. Most of the conventional drugs used for the treatment of RA have severe adverse reactions and are quite expensive. Over the years, increasing proportion of patients with RA and other immune disorders are resorting to complementary and alternative medicine (CAM) for their health needs. Natural plant products comprise one of the most popular CAM for inflammatory and immune disorders. These herbal CAM belong to diverse traditional systems of medicine, including traditional Chinese medicine, Kampo, and Ayurvedic medicine. In this paper, we have outlined the major immunological pathways involved in the induction and regulation of autoimmune arthritis and described various herbal CAM that can effectively modulate these immune pathways. Most of the information about the mechanisms of action of herbal products in the experimental models of RA is relevant to arthritis patients as well. The study of immunological pathways coupled with the emerging application of genomics and proteomics in CAM research is likely to provide novel insights into the mechanisms of action of different CAM modalities.

## 1. Herbal CAM for the Treatment of Inflammatory Autoimmune Arthritis

Conventional (allopathic) anti-inflammatory drugs are the mainstay of treatment for a variety of immune disorders, including rheumatoid arthritis (RA) [1–5]. The nonsteroidal anti-inflammatory drugs (NSAIDs) and biologics (e.g., antitumor necrosis factor (TNF)-*α* antibody and the decoy TNF-*α* receptor) represent a prominent group of such drugs. However, the usage of these drugs is associated with severe adverse effects, including gastrointestinal bleeding and cardiovascular complications [3, 5, 6]. Owing to the side effects and the high cost of conventionally used anti-inflammatory drugs, patients with arthritis are increasingly using complementary and alternative medicine (CAM) modalities of treatment [7–21]. Over 36% Americans used CAM products annually for different disorders and the trend is on the rise [11, 22–25]. Traditional Chinese medicine, Ayurvedic medicine, Kampo, and Homeopathy are among the major contributors to the natural products consumed by patient populations. However, despite the increasing usage and popularity of CAM products in the western world [11, 22–26], one of the main limitations of their use is the meager information about their mechanisms of action and objectivity in evaluating efficacy [27, 28]. This also is one of the main reasons for skepticism about CAM in the minds of both the lay public and the professionals [25, 29–31]. Thus, there is a need for continued studies on the mechanistic aspects of action of CAM products. 

**Figure 1:**
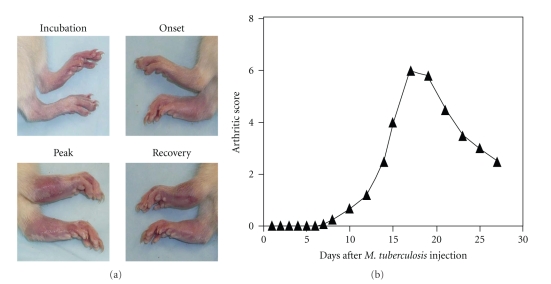
Experimentally-induced adjuvant arthritis (AA) in the Lewis rat. (a) Arthritic paws and (b) the course of the disease. AA is induced by subcutaneous immunization with heat-killed *M. tuberculosis* H37Ra (1 mg/rat) [32–34]. The phases of AA are as follows: incubation, onset, peak, and recovery. The Arthritic Score represents the severity of arthritis [35]. Each paw is graded on a scale of 0 to 4, and total score per rat is derived by adding the scores of all 4 paws of that rat [32–34].

**Figure 2:**
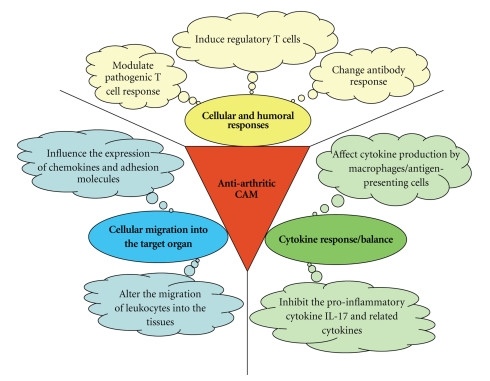
A schematic overview of the immunological effector mechanisms that mediate the antiarthritic activity of different herbal CAM modalities. The herbal products influence the number and/or activity of specific immune mediators (e.g., T cells, antibodies, cytokines, and chemokines), which in turn drive the 3 major immune pathways leading to pathological damage observed in arthritis [5, 43, 44]. These pathways include cellular and humoral immune responses, cytokine response/balance, and cell migration. The net effect of these immunological changes induced by herbal treatment is the suppression of inflammatory and related arthritic processes [5, 43, 44]. The names and geographical origin of specific plant products that induce these changes are listed in Table 1.

**Figure 3:**
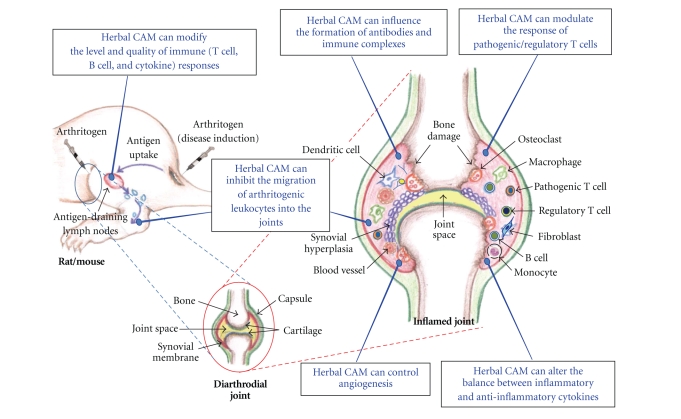
Herbal CAM can intervene at multiple steps in the pathogenesis of autoimmune arthritis. Experimental arthritis can be induced in susceptible rodent strains by subcutaneous (s.c.), intradermal (i.d.), or intraarticular injection of an arthritogen (e.g., Mtb, type II collagen, streptococcal cell wall, etc.). The antigens that are injected s.c. or i.d. are directed into the draining lymph nodes where the immune responses involving the antigen-presenting cells, T cells, and B cells are initiated. The activated lymphocytes and other leukocytes then migrate into the joints and initiate arthritic inflammation via a variety of soluble mediators, including proinflammatory cytokine and antibodies (Figure 1). Herbal CAM can inhibit the initiation and progression of inflammatory arthritis by influencing multiple pathways involved in the disease process. Specific herbs that interfere with particular immune pathway are described in Table 1.

A diverse group of diseases is characterized by inflammation that can be triggered not only by foreign microbial antigens but also by self-antigens. The response to self-antigens results in autoimmune inflammation. Therefore, like the infectious diseases, the autoimmune diseases (such as multiple sclerosis (MS), type-1 diabetes mellitus (T1D), RA, and atherosclerosis) are also associated with inflammation. Considering that autoimmune diseases result from a dysregulated immune system [36, 37], it is imperative to examine and unravel the immunological basis of the therapeutic activity of CAM products against autoimmune disorders as well as other conditions involving inflammation [27, 28, 38–42]. This paper is focused on the immunomodulation of autoimmune arthritis by herbal CAM products. We have described here in detail adjuvant arthritis (AA) (Figure 1) as a prototypic experimental model of RA. Conceptually, the main immune effector pathways in AA are broadly representative of various other animal models of arthritis, for example, collagen-induced arthritis (CIA), streptococcal cell wall-induced arthritis (SCWIA), and proteoglycan-induced arthritis. We have elaborated upon specific immune pathways in arthritis that are modulated by a variety of herbal preparations originating from plants native to different regions of the world (Table 1, and Figures 2 and 3). These immune mechanisms include the cellular and humoral responses, the cytokine response/balance, and the cellular migration into the target organ. 

The above-mentioned immunological events in the pathogenesis of arthritis also offer many promising targets for therapeutic intervention (Figures 2 and 3). We recommend that these and other customized immune parameters be considered for testing besides various biochemical and pharmacological parameters for the evaluation of the mechanisms of action of herbal CAM products. The herbal CAM shown in Table 1 are representative of those tested for the modulation of immunological events that contribute to their antiarthritic activity in vivo in experimental models of arthritis. Understandably, there are several other natural products that possess antiarthritic activity, but their effects on the immune system have not yet been tested. Given the scope of this paper on immune modulation, we have excluded most of those products from Table 1.

## 2. Rheumatoid Arthritis

 RA is prevalent (0.8%) throughout the world and affects all races [5, 84]. Women are affected approximately three times more often than men. The age of onset is between late twenties and early fifties, but no age is immune to the disease. In children and young adults, the disease manifests as juvenile chronic arthritis (JCA). RA is a chronic multisystem disease characterized by persistent inflammatory synovitis usually involving peripheral joints in a symmetric distribution [5, 85]. The synovial inflammation, if uncontrolled, may lead to cartilage damage, bone erosions, and ankylosis of the affected joints [5]. Twin studies and family studies indicate that there is a genetic predisposition to RA [86], and about 70% of patients have HLA-DR4 or -DR1 alleles or both. The precise target autoantigen for RA has not yet been identified. Type II collagen (CII), aggrecan, immunoglobulin binding protein (BiP), and heat-shock protein 65 (Hsp65) are among the antigens that have been implicated in the pathogenesis of RA [5, 85]. As mentioned above, NSAIDS are the mainstay of therapy for a large proportion of patients with RA. However, because of adverse reactions, high costs, and limited efficacy of these drugs in many patients [3–5], the use of CAM by RA patients is becoming increasingly popular in USA and other developed countries [9, 10, 13, 14, 16, 20].

## 3. Adjuvant Arthritis: An Experimental Model of RA

 AA can be induced in the Lewis (RT.1^l^) rat by immunization with heat-killed *Mycobacterium tuberculosis *(Mtb) (H37Ra) [87]. The disease manifests as inflammation of the paws including the paw joints. The paw inflammation affects primarily the ankles, wrists, and smaller joints. The arthritic inflammation starts after 8–10 days, peaks between day 15–17, and then undergoes a spontaneous, gradual recovery in the subsequent 12–15 days (Figure 1). The primary immune reaction in paw joints is the mononuclear cell infiltration of the synovial tissue, which if uncontrolled, can lead to damage to cartilage and bone [87]. Mycobacterial hsp65 (Bhsp65) has been invoked in the pathogenesis of AA. Following Mtb injection, Bhsp65 is taken up by the regional draining lymph nodes where antigen-presenting cells (APCs) process and present this antigen to naïve T cells (Figure 3). The T cells bearing receptors specific for epitopes within Bhsp65 then get activated and undergo proliferation. These antigen-primed T cells then leave the lymph nodes to enter into the peripheral circulation. These T cells then migrate out from the blood vessels into the target organ, the joint, where they initiate the immune pathology (Figure 3). Rat AA shares several features with human RA, and thereby, it serves as an excellent model for RA [87]. 

The AA model has extensively been used for studies regarding the pathogenesis of autoimmune arthritis [32, 43, 88, 89] as well as for the testing of new natural [33, 34, 58] or synthetic antiarthritic therapeutic products. A variety of herbal CAM products have been shown to attenuate the severity of the disease in the rat AA model (Table 1). These herbs modulate different immunological effector and regulatory pathways (discussed below in detail) involved in the disease process (Figures 2 and 3). Another model of chronic inflammation leading to bone loss has also been employed to examine the role of natural products (e.g., green tea) in limiting bone damage and bone loss, which accompanies chronic arthritis [90, 91].

## 4. Heat-Shock Proteins (Hsps) Serve as the Target Antigens in Autoimmune Arthritis

 Hsps have been associated with many autoimmune diseases such as RA, Crohn's disease, MS, and systemic lupus erythematosus (SLE) [92]. Hsps may also induce protection against arthritis [93]. Most hsps are acute stress reactants that insure cell survival under hostile conditions. They also are the molecular chaperones involved in protein folding and other functions for maintaining the structural integrity of other proteins [92]. A T-cell clone that was arthritogenic for the Lewis rat was found to be specific for the epitope 180–188 (p180–188) of Bhsp65 [88]. In juvenile chronic arthritis (JCA) patients, there was T-cell reactivity to p180–188 of Bhsp65, as well as to the partially homologous determinant within articular cartilage link protein [94]. The T cells of these patients also showed significant response to human hsp60 [95] and Bhsp65 [94, 96], emphasizing the importance of Hsp65 as one of the major antigens in arthritis pathogenesis. Other hsps, including hsp70 and hsp47 have also been invoked in the pathogenesis of AA [97]. Similarities in immune response to disease-related antigens in rodent models and humans have provided useful insights into the disease pathogenesis. 

It has been shown that natural plant products such as flavonoids and Celastrol can alter the cellular expression of hsps [98–100]. These observations are potentially of significance in view of the observed disease-protective effects of green tea flavonoids [33] and the ethanol extract of Celastrus [58]. However, the precise mechanistic link between the two sets of observations remains to be fully defined. In regard to the modulation of antigen-specific T-cell response to hsps by CAM modalities, we have shown that feeding of the Chinese herbal formula Huo Luo Xiao Ling (HLXL) dan to Lewis rats significantly reduced the T-cell proliferative response to Bhsp65 [34], and this effect was associated with suppression of arthritis. On the contrary, feeding of rats with green tea extract [33] or Celastrus [58] failed to influence T-cell proliferative response to Bhsp65 despite significant reduction in the severity of AA. However, as described below, these rats showed significant changes in cytokine response to Bhsp65 but without any change in T-cell proliferation, revealing a dichotomy of the two immune parameters. In support of our results, several other natural products (e.g., Quercetin, Resveratrol, Kaempferol, Vineatrol) have also been reported to influence lymphocyte proliferative responses, with most of them leading to inhibition of proliferative response [101–103]. Also reported is the dissociation between proliferation and function of lymphocytes exposed to natural products like Kaemferol [103].

## 5. CD4+CD25+ T-Regulatory Cells (Treg) Are Vital for Self-Tolerance and Regulation of Autoimmunity

 Many types of regulatory T cells, including Th2, Th3, Treg, NKT cells, and Tr1 have been described [104]. The most recent addition to the group of regulatory T cells is the CD4+CD25+ T-regulatory cell (Treg) [105–107]. Treg have emerged as the central controllers of autoimmunity in a variety of experimental models of human autoimmune diseases [105–107]. Importantly, in animal models, CD4+CD25+ T-cell therapy via adoptive transfer of cells can effectively delay and suppress a variety of immunological diseases including diabetes, colitis, and gastritis [105–107]. On the contrary, the in vivo depletion of Treg leads to the early initiation and/or aggravation of autoimmune arthritis [106–109] as well as other autoimmune diseases. 

There are two distinct types of Treg: the “natural Treg” that developed in the thymus and the “adaptive (induced) Treg” that developed in the periphery in response to antigen exposure [106]. The mechanism of action of Treg involves cell-cell contact between Treg and the responder cells, and it requires activation of Treg via the T-cell receptor (TCR) [105–107]. Secreted TGF-*β* and IL-10 have been suggested to mediate suppression by Treg in vivo. 

There is evidence for a reciprocal control of the differentiation of T helper 17 (Th17) and Treg. The differentiation of the proinflammatory and pathogenic Th17 cells is induced by the simultaneous presence of TGF-*β* and IL-6, whereas the presence of TGF-*β* alone induces the generation of Treg expressing the transcription factor Foxp3 [110]. 

Over the last 5–10 years, the significance of determining the frequency and suppressive function of Treg for evaluating the autoimmune disease process as well as assessing the efficacy of therapeutic products for different autoimmune diseases is increasingly being realized [105–107, 111]. Defects in Treg have been reported in RA [112], and this reduced activity of Treg can be restored following successful therapy, for example, with anti-TNF-*α* in the case of RA [112]. A recent study in the area of transplantation research has shown that dendritic cells treated with triptolide (derived from the Chinese herb *Tripterygium wilfordii*) promotes the expansion of Treg in vitro [57]. It is hoped that assessment of Treg number and function would be incorporated regularly in studies aimed at defining the mechanisms of action of various CAM modalities, including natural plant products. Also, as described above, there are various other types of regulatory T cells besides Treg [104]. It is likely that different CAM modalities might have differential effect on distinct subsets of regulatory T cells, such that one product may have a more pronounced effect on Treg, while the other might instead have a major effect on Th2 or Tr1 type of regulatory cells.

## 6. Antibodies Contribute to the Pathogenesis of Autoimmune Arthritis

 Studies in a spontaneous model of autoimmune arthritis have underscored the importance of antibodies in mediating the immune pathology in this disease; the pathology is initiated by T cells but subsequently perpetuated by antibodies [113]. Studies in the CIA model in mice have clearly demonstrated the importance of antibodies to type II collagen (CII) in the disease process [114]. However, at this time there is not much information about the physiologic role of antibodies to hsp65 in AA. The pathogenic effect of anti-Bhsp65 antibodies in AA has not been excluded formally. On the contrary, there is evidence from work done by other investigators and us [35, 115] pointing to the protective effect against AA of anti-Bhsp65 antibodies. In one study, the AA-protective effect of anti-Bhsp65 antibodies was attributed to the production of IL-10 from mononuclear cells [115]. 

In the CIA model, extracts of green tea [59] pomegranate [60], and Taxol [63, 64] have been shown to suppress arthritis, and this effect was associated with a significant decrease in anti-CII antibodies. A similar effect on the clinical disease, the proinflammatory cytokines, and the serum IgG2a was reported in another study on CIA following treatment with curcumin, a major component of turmeric [61]. Turmeric extract has also been shown to induce protection against arthritis in streptococcal cell wall-induced arthritis model of RA [78]. In one of our studies based on the AA model, we observed that feeding the polyphenolic extract of green tea to Lewis rats resulted in a significant decrease in the antibody response to Bhsp65 [33]. Similar results were obtained with a traditional Chinese medicine, HLXL, which is a mixture of 11 different herbs [34]. In both cases, the decrease in antibody response was associated with a corresponding reduction in the severity of arthritis. However, not all antiarthritic herbs tested by us caused a decrease in antibody response to Bhsp65. In another study, we observed the opposite as the feeding of Celastrus to Lewis rats led to an increased anti-Bhsp65 antibody response despite a significant suppression of clinical arthritis [58]. 

At present, we do not have additional information to clarify the differences in the functional attributes of the antibody subsets that are predominantly altered following feeding of different plant products. However, we propose that anti-Bhsp65 antibodies produced during the course of AA in the Lewis rats belong to two main categories, pathogenic and protective [35]. In this context, we suggest that different herbs target distinct subsets of antibodies such that reduction in clinical arthritis might involve either the suppression of pathogenic antibodies or the enhancement of protective antibodies, or both. Furthermore, studies of antibody patterns using a panel of antigens [40, 41] targeted in arthritis and other autoimmune diseases might provide a useful readout for the effect of CAM products on the disease process.

## 7. Regulation of Autoimmunity via T-Helper (Th1)-/Th2-Type Cytokine Balance

 Proinflammatory cytokines TNF-*α*, IL-1*β*, and IL-6 produced by macrophages and other immune cells are of critical importance in the initiation and propagation of arthritis [116, 117]. Among the T cell, the Th1 cells secrete IFN-*γ* and TNF-*α*, whereas the counter-regulatory Th2 cells secrete IL-4, IL-5, IL-10, and IL-13 [118]. Th1 cells are primarily involved in the pathogenesis of certain organ-specific autoimmune diseases, whereas Th2 cells play a major role in systemic autoimmunity. The role of Th1-Th2 balance in regulation of autoimmunity has been validated through several animal model studies. The susceptibility or resistance to disease [119] and protection from disease [120], as well as improvement of the disease in RA patients [117] was associated with a change in cytokine balance to Th2 type. The change in Th1/Th2 balance could occur either by a decrease in the proinflammatory cytokine (e.g., IFN-*γ*) or an increase in the anti-inflammatory cytokine (e.g., IL-4/IL-10), or both [42, 118]. 

In a study on CIA, activation of the Th2 response was shown to inhibit IFN-*γ* production as well as reduction in the severity of arthritis [121]. Other investigators have reported the downmodulation of CIA coupled with suppression of proinflammatory cytokines (e.g., TNF-*α*, IL-1*β*, and IL-6) by treatment of mice with extracts of green tea [59], pomegranate [60], or *Plectranthus amboinicus* [68]. A similar effect has been observed in vitro with Moutan cortex [122]. In 3 separate studies in AA using different natural plant products, namely, Celastrus [58], green tea [33], and HLXL [34], we observed that each of these three herbal products induced protection against AA coupled with an altered Th1/Th2 ratio. The latter effect was caused primarily by an increase in IL-10 while IFN-*γ* remained unchanged. Enigmatically, it has also been observed that proinflammatory Th1 cytokines such as IFN-*γ* and TNF-*α* might display dual roles as inflammatory and immunosuppressive cytokines [123]. For example, the suppression of inflammation by IFN-*γ* has been observed in AA [123]. Therefore, herbal CAM-induced changes in the level of certain cytokines with dual functions need to be evaluated with caution.

## 8. T-Helper 17 (Th17) Cells Mediate Inflammation and Tissue Damage in Arthritis

 Th17 cells secrete IL-17, which has been shown to be involved in inflammatory and autoimmune diseases [110, 124]. Th17 subset of T cells is distinct from Th1 and Th2 cells, and the differentiation of Th17 cells is induced by the concurrent exposure to TGF-*β* and IL-6 [110]. Retinoic acid-related orphan receptor gamma-t (ROR*γ*t) is the transcription factor required for the differentiation of Th17 cells. IFN-*γ*, IL-2, IL-4, and IL-27 have been shown to inhibit the activity of Th17 cells, whereas IL-21 and IL-23 are important for the clonal expansion and stabilization (maintenance) of Th17 cells [110]. IL-17 has been implicated in the pathogenesis of autoimmune diseases including arthritis [125]. Abundant quantities of IL-17 have been found in the synovial fluid of RA patients [125]. The *in vivo* blockade of IL-17 by soluble IL-17 receptors or by neutralizing anti-IL-17 antibody can significantly attenuate arthritis in rodents [126]. Furthermore, mice deficient in IL-17 [127] or IL-17 receptor [128] were found to be resistant to the induction of CIA. 

In the preceding section, we have summarized the results of our earlier studies showing that a shift in Th1 to Th2 ratio induced by natural plant products was associated with reduced severity of AA in Lewis rats [33, 34, 58]. In two of these studies, we also tested the IL-17 response. Importantly, feeding rats with green tea [33] or HLXL [34] led to a significant reduction in IL-17 response. Thus, the concurrent changes in Th1/Th2 ratio and IL-17 response culminated into a beneficial antiarthritic activity of green tea and HLXL.

## 9. Chemokines and Adhesion Molecules Orchestrate the Migration of Leukocytes into the Target Organ in Arthritis

 The migration of lymphocytes, macrophages, and other cells from blood into the joints is orchestrated by defined interactions mediated by chemokines and adhesion molecules [129, 130]. Chemokines are chemoattractant cytokines that direct the migration of leukocytes from blood vessel lumen into the target site of inflammation in the periphery. The expression of chemokines and their receptors is influenced by cytokines and other inflammatory mediators. Dysregulated expression of chemokines and/or their receptors may lead to immune pathology. The blocking or neutralization of these molecules via antagonists or antibodies is being explored for the treatment of arthritis in experimental models [131, 132] and RA patients [133, 134]. Thus, study of the levels of expression of different chemokines and adhesion molecules, and the blockade of these biomolecules by appropriate reagents can serve as an important tool for defining the mechanisms of actions of CAM products that have antiarthritic activity. 

Many herbal products have been reported to modulate the expression of specific chemokines in different tissues [135–142], and many of these chemokines are relevant for the trafficking of leukocytes into the joints in arthritis as well [129, 130]. In one of our studies, we reported a simple method to study the in vivo migration of radiolabeled leukocytes in vivo [44]. We also showed a clear association between the kinetics of migration of leukocytes through the joints and the susceptibility to AA [44]. The radiolabel can be replaced by a fluorescent dye as needed for future use of such assays in CAM studies.

## 10. Concluding Remarks

This paper is focused on cellular and humoral immunological effector mechanisms that mediate the action of a wide variety of herbal CAM for the treatment of experimental autoimmune arthritis. However, natural products can contribute to the suppression of inflammation and arthritic processes via altering specific molecular mediators of these pathways. For example, the antiarthritic activity of various compounds (Tea polyphenols, Boswellic acid, Morin, etc.) purified from natural products has been attributed in part to their anti-oxidant activity [33, 59] and to their action on nuclear factor-kB (NF-kB), cyclooxygenase-2 (COX-2), 5-lipoxygenase (5-LOX), and matrix metalloproteinases (MMPs) (reviewed in [18]). Therefore, future studies on herbal products would benefit from including test parameters that span across pathological, immunological, biochemical, and molecular biology-related aspects of the disease process. For immunological aspects, we hope to see more CAM studies both in vitro and in vivo on the newer cytokines (e.g., the IL-17/IL-23 axis) and Treg. Furthermore, the study of genomics and proteomics of CAM [143–145] is representative of several modern research tools whose investment in CAM research is currently underway. This in turn would not only enhance the depth and scope of investigations into CAM research, but also provide an interface where CAM and conventional medicine could find a common ground for understanding the mechanisms of action of therapeutic products and their practical use for the ultimate benefit of the patients. It is rather difficult to predict with certainty the natural products or compounds that might end up as successful therapeutic agents for RA. Nevertheless, on the basis of the results obtained from animal models of RA as well as the delineation of multiple immunological and molecular targets of the indicated herbal products, we find Tea polyphenols, Celastrol, Triptolide, Curcumin, Boswellic acids, and HLXL as promising candidates for further preclinical and clinical trials in RA.

## Figures and Tables

**Table 1:** Mechanisms of immunomodulation by herbal products.

Herbs	Origin	Reference
(A) **Cellular and humoral responses**		

*(A.1) Effect on T cell response (T cell activation, T cell proliferation, ratio of CD* ^a^ *4/CD8 cells, etc.)*		
*Pterodon pubescens*	Brazil	[45]
*Chrysanthemum indicum, *Fumigant I^b^, Huo-Luo-Xiao-Ling Dan^b^, *Litsea coreana, Radix Linderae, Tripterygium wilfordii *	China	[34, 46–52]
Dai-bofu-to^b^, *Stephania tetrandra *	Japan	[53, 54]
*Centella asiatica *	Southeastern Asia/China	[55]

*(A.2) Induction/expansion of regulatory T cells*		
*Chelidonium majus*	Korea	[56]
Triptolide (*Tripterygium wilfordii*)	China	[57]

*(A.3) Change in antibody/B cell response*		
*Pterodon pubescens*	Brazil	[45]
*Camellia sinensis, *Curcumin, *Celastrus aculeatus,* Huo-Luo-Xiao-Ling-Dan^b^, Pomegranate extract, *Radix Linderae *	China, Korea	[33, 34, 50, 58–61]
*Stephania tetrandra*	Japan	[54]
*Barrington racemosa*	India	[62]
*Centella asiatica*	Southeastern Asia/China	[55]
*Taxus brevifolia, Fumagillin analogue *	North America	[63, 64]

(B) **Cytokine response/balance**		

*(B.1) Affecting major cytokines produced by macrophages/antigen-presenting cells (TNF-*α*, IL-1, IL-6, etc.) and/or deviation of the response to Th2 type*		
*Nyctanthes arbor-tristis, Swertia chirayita*	India	[65, 66]
*Zingiber officinale*	India/China	[67]
*Boswellia carterii, Camellia sinensis,* Cherries^b^, Fumigant I^b^, Curcumin, Huo Luo-Xiao-Ling-Dan^b^, *Litsea coreana, Paeonia lactiflora, Plectranthus amboinicus,* Pomegranate extract,* Sinomenium acutum*, QFGJS^b^, *Tripterygium wilfordii, Turpinia Arguta *	China, Korea, India	[47–49, 51, 52, 59–61, 68–75]
*Chelidonium majus, *PG201, *Ulmus davidiana *	Korea	[56, 76, 77]

*(B.2) Inhibiting the pathogenic cytokine IL-17 and related cytokines*		
*Camellia sinensis, *Huo-Luo-Xiao-Ling-Dan^b^, *Tripterygium wilfordii *	China	[33, 34, 52, 59]

(C) **Cellular migration into the target organ**		

*(C.1) Affecting the expression of chemokines and adhesion molecules in the blood vessels or joint tissues*		
Fumigant I^b^	China	[49]

*(C.2) Altering the migration of leukocytes into the tissues-monocytes, macrophages, neutrophils, lymphocytes, etc.*		
*Curcuma longa*	India/China	[78, 79]
*Pterodon pubescens*	Brazil	[45]
*Camellia sinensis*	China	[59]
*Centella asiatica*	Southeastern Asia/China	[55]

(D) **Mechanism of action not yet determined**		

Bai jiang cao^b^, Duhuo^b^, Sanqui^b^, Yan hu suo^b^	China	[80]
*Chlorophytum borivilianum, Ocimum sanctum*	India	[81, 82]
Shu-Jing-Huo-Xue-Tang^b^	Japan	[83]

The mechanisms of immunomodulation by herbs were studied using various experimental rodent models of human rheumatoid arthritis, for example, adjuvant arthritis (AA), Collagen-induced arthritis (CIA), and streptococcal cell wall-induced arthritis (SCWIA).

^a^CD: Cluster of differentiation; IL: Interleukin; TNF-*α*: Tumor-necrosis factor-alpha.

^b^Herbal mixtures.

## References

[1] Kwoh CK, Simms RW, Anderson LG (1996). Guidelines for the management of rheumatoid arthritis: American College of Rheumatology Ad Hoc Committee on Clinical Guidelines. *Arthritis and Rheumatism*.

[2] Simon LS, Yocum D (2000). New and future drug therapies for rheumatoid arthritis. *Rheumatology*.

[3] Kremers HM, Nicola P, Crowson CS, O’Fallon WM, Gabriel SE (2004). Therapeutic strategies in rheumatoid arthritis over a 40-year period. *Journal of Rheumatology*.

[4] Olsen NJ, Stein CM (2004). New Drugs for Rheumatoid Arthritis. *The New England Journal of Medicine*.

[5] Lipsky PE, Kasper DL, Braunwald E, Fauci AS, Hauser SL, Longo DL, Jameson JL (2005). Rheumatoid arthritis. *Harrison’s Principles of Internal Medicine*.

[6] Couzin J (2004). Withdrawal of Vioxx casts a shadow over COX-2 inhibitors. *Science*.

[7] Lipsky PE, Tao X-L (1997). A potential new treatment for rheumatoid arthritis: thunder god vine. *Seminars in Arthritis and Rheumatism*.

[8] Astin JA, Beckner W, Soeken K, Hochberg MC, Berman B (2002). Psychological interventions for rheumatoid arthritis: a meta-analysis of randomized controlled trials. *Arthritis Care and Research*.

[9] Cibere J, Deng Z, Lin Y (2003). A randomized double blind, placebo controlled trial of topical Tripterygium wilfordii in rheumatoid arthritis: reanalysis using logistic regression analysis. *Journal of Rheumatology*.

[10] Taibi DM, Bourguignon C (2003). The role of complementary and alternative therapies in managing rheumatoid arthritis. *Family & Community Health*.

[11] Barnes PM, Powell-Griner E, McFann K, Nahin RL (2004). Complementary and alternative medicine use among adults: United States, 2002. *Advance Data*.

[12] Soeken KL (2004). Selected CAM therapies for arthritis-related pain: the evidence from systematic reviews. *Clinical Journal of Pain*.

[13] Ahmed S, Anuntiyo J, Malemud CJ, Haqqi TM (2005). Biological basis for the use of botanicals in osteoarthritis and rheumatoid arthritis: a review. *Evidence-Based Complementary and Alternative Medicine*.

[14] Zhang GG, Lee W, Bausell B, Lao L, Handwerger B, Berman B (2005). Variability in the Traditional Chinese Medicine (TCM) diagnoses and herbal prescriptions provided by three TCM practitioners for 40 patients with rheumatoid arthritis. *Journal of Alternative and Complementary Medicine*.

[15] Gagnier JJ, van Tulder M, Berman B, Bombardier C (2006). Herbal medicine for low back pain. *Cochrane Database of Systematic Reviews*.

[16] Nozaki K, Hikiami H, Goto H, Nakagawa T, Shibahara N, Shimada Y (2006). Keishibukuryogan (Gui-Zhi-Fu-Ling-Wan), a Kampo formula, decreases disease activity and soluble vascular adhesion molecule-1 in patients with rheumatoid arthritis. *Evidence-Based Complementary and Alternative Medicine*.

[17] Hijikata Y, Miyamae Y, Takatsu H, Sentoh S (2007). Two Kampo medicines, Jidabokuippo and Hachimijiogan alleviate sprains, bruises and arthritis. *Evidence-Based Complementary and Alternative Medicine*.

[18] Khanna D, Sethi G, Ahn KS (2007). Natural products as a gold mine for arthritis treatment. *Current Opinion in Pharmacology*.

[19] Chen KW, Perlman A, Liao JG, Lam A, Staller J, Sigal LH (2008). Effects of external qigong therapy on osteoarthritis of the knee. *Clinical Rheumatology*.

[20] Sengupta K, Alluri KV, Satish AR (2008). A double blind, randomized, placebo controlled study of the efficacy and safety of 5-Loxin for treatment of osteoarthritis of the knee. *Arthritis Research and Therapy*.

[21] Callahan LF, Wiley-Exley EK, Mielenz TJ (2009). Use of complementary and alternative medicine among patients with arthritis. *Preventing Chronic Disease*.

[23] Eisenberg DM, Davis RB, Ettner SL (1998). Trends in alternative medicine use in the United States, 1990–1997: results of a follow-up national survey. *Journal of the American Medical Association*.

[24] Kuo GM, Hawley ST, Weiss LT, Balkrishnan R, Volk RJ (2004). Factors associated with herbal use among urban multiethnic primary care patients: a cross-sectional survey. *BMC Complementary and Alternative Medicine*.

[25] Manheimer E, Berman B (2003). NCCAM support for the cochrane collaboration CAM field. *Complementary Therapies in Medicine*.

[26] Crawford NW, Cincotta DR, Lim A, Powell CVE (2006). A cross-sectional survey of complementary and alternative medicine use by children and adolescents attending the University Hospital of Wales. *BMC Complementary and Alternative Medicine*.

[27] Cooper EL, Hecht SB (2005). Latin American Center symposium on environment and health: exploring natural products—April 19, 2005, Moss auditorium, room A2-342 Marion Davies Children’s Clinic (MDCC) Center for Health Sciences. *Evidence-Based Complementary and Alternative Medicine*.

[28] Cooper EL (2007). The immune system and complementary and alternative medicine. *Evidence-Based Complementary and Alternative Medicine*.

[29] Berman BM, Singh BK, Lao L, Singh BB, Ferentz KS, Hartnoll SM (1995). Physicians’ attitudes toward complementary or alternative medicine: a regional survey. *The Journal of the American Board of Family Practice*.

[30] Ben-Arye E, Frenkel M, Klein A, Scharf M (2008). Attitudes toward integration of complementary and alternative medicine in primary care: perspectives of patients, physicians and complementary practitioners. *Patient Education and Counseling*.

[31] Desylvia D, Stuber M, Fung CC, Bazargan-Hejazi S, Cooper E The knowledge, attitudes and usage of complementary and alternative medicine of medical students.

[32] Durai M, Kim HR, Bala K, Moudgil KD (2007). T cells against the pathogenic and protective epitopes of heat-shock protein 65 are crossreactive and display functional similarity: novel aspect of regulation of autoimmune arthritis. *Journal of Rheumatology*.

[33] Kim HR, Rajaiah R, Wu Q-L (2008). Green tea protects rats against autoimmune arthritis by modulating disease-related immune events. *Journal of Nutrition*.

[34] Rajaiah R, Lee DY-W, Ma Z (2009). Huo-Luo-Xiao-Ling Dan modulates antigen-directed immune response in adjuvant-induced inflammation. *Journal of Ethnopharmacology*.

[35] Kim HR, Kim EY, Cerny J, Moudgil KD (2006). Antibody responses to mycobacterial and self heat shock protein 65 in autoimmune arthritis: epitope specificity and implication in pathogenesis. *The Journal of Immunology*.

[36] Rose NR (2002). Mechanisms of autoimmunity. *Seminars in Liver Disease*.

[37] Atassi MZ, Casali P (2008). Molecular mechanisms of autoimmunity. *Autoimmunity*.

[38] Kasahara S, Cooper EL (2004). Nervous, endocrine, immune systems as a target for complementary and alternative medicine. *Advances in Experimental Medicine and Biology*.

[39] Franceschi C (2007). Inflammaging as a major characteristic of old people: can it be prevented or cured?. *Nutrition Reviews*.

[40] Vojdani A (2008). Antibodies as predictors of complex autoimmune diseases. *International Journal of Immunopathology and Pharmacology*.

[41] Vojdani A, Bazargan M, Vojdani E (2004). Heat shock protein and gliadin peptide promote development of peptidase antibodies in children with autism and patients with autoimmune disease. *Clinical and Diagnostic Laboratory Immunology*.

[42] Watanabe T, Yamamoto T, Yoshida M (2009). The traditional herbal medicine saireito exerts its inhibitory effect on murine oxazolone-induced colitis via the induction of Th1-polarized immune responses in the mucosal immune system of the colon. *International Archives of Allergy and Immunology*.

[43] Shahrara S, Proudfoot AEI, Woods JM (2005). Amelioration of rat adjuvant-induced arthritis by Met-RANTES. *Arthritis and Rheumatism*.

[44] Mia MY, Kim EY, Satpute SR, Moudgil KD (2008). The dynamics of articular leukocyte trafficking and the immune response to self heat-shock protein 65 influence arthritis susceptibility. *Journal of Clinical Immunology*.

[45] Coelho MGP, Sabino KCC, Dalmau SR (2004). Immunomodulatory effects of sucupira (Pterodon pubescens) seed infusion on collagen-induced arthritis. *Clinical and Experimental Rheumatology*.

[46] Chen X-Y, Li J, Cheng W-M, Jiang H, Xie X-F, Hu R (2008). Effect of total flavonoids of chrysanthemum indicum on the apoptosis of synoviocytes in joint of adjuvant arthritis rats. *American Journal of Chinese Medicine*.

[47] Ramgolam V, Ang SG, Lai YH, Loh CS, Yap HK (2000). Traditional Chinese medicines as immunosuppressive agents. *Annals of the Academy of Medicine Singapore*.

[48] Setty AR, Sigal LH (2005). Herbal medications commonly used in the practice of rheumatology: mechanisms of action, efficacy, and side effects. *Seminars in Arthritis and Rheumatism*.

[49] Shen Y, Lu J-D (2009). Treatment of adjuvant arthritis in rats with Chinese herbal fumigation: efficacy and mechanism. *Journal of Chinese Integrative Medicine*.

[50] Wang C, Dai Y, Yang J, Chou G, Wang C, Wang Z (2007). Treatment with total alkaloids from Radix Linderae reduces inflammation and joint destruction in type II collagen-induced model for rheumatoid arthritis. *Journal of Ethnopharmacology*.

[51] Wang T-Y, Li J, Ge J-F (2008). Preliminary study of total flavonoids from Litsea coreana Levl. on experimental adjuvant-induced arthritis in rats. *American Journal of Chinese Medicine*.

[52] Wang Y, Jia L, Wu C-Y (2008). Triptolide inhibits the differentiation of Th17 cells and suppresses collagen-induced arthritis. *Scandinavian The Journal of Immunology*.

[53] Inoue M, Ono Y, Mizukami H (2004). Suppressive effect of dai-bofu-to on collagen-induced arthritis. *Biological and Pharmaceutical Bulletin*.

[54] Niizawa A, Kogure T, Hai LX (2003). Clinical and immunomodulatory effects of Fun-boi, an herbal medicine, on collagen-induced arthritis in vivo. *Clinical and Experimental Rheumatology*.

[55] Liu M, Dai Y, Yao X (2008). Anti-rheumatoid arthritic effect of madecassoside on type II collagen-induced arthritis in mice. *International Immunopharmacology*.

[56] Lee Y-C, Kim S-H, Roh S-S, Choi H-Y, Seo Y-B (2007). Suppressive effects of Chelidonium majus methanol extract in knee joint, regional lymph nodes, and spleen on collagen-induced arthritis in mice. *Journal of Ethnopharmacology*.

[57] Liu Y, Chen Y, Lamb JR, Tam PKH (2007). Triptolide, a component of Chinese herbal medicine, modulates the functional phenotype of dendritic cells. *Transplantation*.

[58] Tong L, Moudgil KD (2007). Celastrus aculeatus Merr. suppresses the induction and progression of autoimmune arthritis by modulating immune response to heat-shock protein 65. *Arthritis Research and Therapy*.

[59] Haqqi TM, Anthony DD, Gupta S (1999). Prevention of collagen-induced arthritis in mice by a polyphenolic fraction from green tea. *Proceedings of the National Academy of Sciences of the United States of America*.

[60] Shukla M, Gupta K, Rasheed Z, Khan KA, Haqqi TM (2008). Consumption of hydrolyzable tannins-rich pomegranate extract suppresses inflammation and joint damage in rheumatoid arthritis. *Nutrition*.

[61] Moon D-O, Kim M-O, Choi YH, Park Y-M, Kim G-Y (2010). Curcumin attenuates inflammatory response in IL-1*β*-induced human synovial fibroblasts and collagen-induced arthritis in mouse model. *International Immunopharmacology*.

[62] Patil KR, Patil CR, Jadhav RB, Mahajan VK, Patil PR, Gaikwad PS Anti-arthritic Activity of Bartogenic Acid Isolated from Fruits of Barringtonia racemosa Roxb. (Lecythidaceae).

[63] Brahn E, Tang C, Banquerigo ML (1994). Regression of collagen-induced arthritis with taxol, a microtubule stabilizer. *Arthritis and Rheumatism*.

[64] Oliver SJ, Banquerigo ML, Brahn E (1994). Suppression of collagen-induced arthritis using an angiogenesis inhibitor, AGM-1470, and a microtubule stabilizer, Taxol. *Cellular Immunology*.

[65] Chevrier MR, Ryan AE, Lee DY-W, Zhongze M, Wu-Yan Z, Via CS (2005). Boswellia carterii extract inhibits TH1 cytokines and promotes TH2 cytokines in vitro. *Clinical and Diagnostic Laboratory Immunology*.

[66] Kumar IV, Paul BN, Asthana R, Saxena A, Mehrotra S, Rajan G (2003). Swertia chirayita Mediated Modulation of Interleukin-1*β*, Interleukin-6, Interleukin-10, Interferon-*γ*, and Tumor Necrosis Factor-*α* in Arthritic Mice. *Immunopharmacology and Immunotoxicology*.

[67] Funk JL, Frye JB, Oyarzo JN, Timmermann BN (2009). Comparative effects of two gingerol-containing zingiber officinale extracts on experimental Rheumatoid arthritis. *Journal of Natural Products*.

[68] Chang J-M, Cheng C-M, Hung L-M, Chung Y-S, Wu R-Y (2010). Potential use of plectranthus amboinicus in the treatment of rheumatoid arthritis. *Evidence-Based Complementary and Alternative Medicine*.

[69] Cai X, Zhou H, Wong YF (2007). Suppression of the onset and progression of collagen-induced arthritis in rats by QFGJS, a preparation from an anti-arthritic Chinese herbal formula. *Journal of Ethnopharmacology*.

[70] Fan AY, Lao L, Zhang RX (2005). Effects of an acetone extract of Boswellia carterii Birdw. (Burseraceae) gum resin on adjuvant-induced arthritis in lewis rats. *Journal of Ethnopharmacology*.

[71] He Y-H, Xiao C, Wang Y-S (2005). Antioxidant and anti-inflammatory effects of cyanidin from cherries on rat adjuvant-induced arthritis. *Zhongguo Zhongyao Zazhi*.

[72] Xu H-M, Wei W, Jia X-Y, Chang Y, Zhang L (2007). Effects and mechanisms of total glucosides of paeony on adjuvant arthritis in rats. *Journal of Ethnopharmacology*.

[73] Yang D-S, Liu F, Zeng F-D, Chen H (2005). Effect of sinomenine on adjuvant arthritis and its mechanisms. *Zhongguo Zhongyao Zazhi*.

[74] Zhang L, Li J, Yu S-C (2008). Therapeutic effects and mechanisms of total flavonoids of Turpinia Arguta Seen on adjuvant arthritis in rats. *Journal of Ethnopharmacology*.

[75] Zhang R-X, Fan AY, Zhou A-N (2009). Extract of the Chinese herbal formula Huo Luo Xiao Ling Dan inhibited adjuvant arthritis in rats. *Journal of Ethnopharmacology*.

[76] Kim K-S, Lee S-D, Kim K-H, Kil S-Y, Chung K-H, Kim C-H (2005). Suppressive effects of a water extract of Ulmus davidiana Planch (Ulmaceae) on collagen-induced arthritis in mice. *Journal of Ethnopharmacology*.

[77] Shin SS, Jin M, Jung HJ (2003). Suppressive effects of PG201, an ethanol extract from herbs, on collagen-induced arthritis in mice. *Rheumatology*.

[78] Funk JL, Oyarzo JN, Frye JB (2006). Turmeric extracts containing curcuminoids prevent experimental rheumatoid arthritis. *Journal of Natural Products*.

[79] Funk JL, Frye JB, Oyarzo JN (2006). Efficacy and mechanism of action of turmeric supplements in the treatment of experimental arthritis. *Arthritis and Rheumatism*.

[80] Wei F, Zou S, Young A, Dubner R, Ren K (1999). Effects of four herbal extracts on adjuvant-induced inflammation and hyperalgesia in rats. *Journal of Alternative and Complementary Medicine*.

[81] Prakash P, Gupta N (2005). Therapeutic uses of Ocimum sanctum Linn (Tulsi) with a note on eugenol and its pharmacological actions: a short review. *Indian Journal of Physiology and Pharmacology*.

[82] Thakur GS, Bag M, Sanodiya BS (2009). Chlorophytum borivilianum: a white gold for biopharmaceuticals and neutraceuticals. *Current Pharmaceutical Biotechnology*.

[83] Kanai S, Taniguchi N, Higashino H (2003). Study of Sokei-Kakketu-To (Shu-Jing-Huo-Xue-Tang) in adjuvant arthritis rats. *American Journal of Chinese Medicine*.

[84] Lawrence RC, Helmick CG, Arnett FC (1998). Estimates of the prevalence of arthritis and selected musculoskeletal disorders in the United States. *Arthritis and Rheumatism*.

[85] Gorman CL, Cope AP (2008). Immune-mediated pathways in chronic inflammatory arthritis. *Best Practice and Research*.

[86] Aho K, Koskenvuo M, Tuominen J, Kaprio J (1986). Occurrence of rheumatoid arthritis in a nationwide series of twins. *Journal of Rheumatology*.

[87] Pearson CM (1956). Development of arthritis, periarthritis and periostitis in rats given adjuvants. *Proceedings of the Society for Experimental Biology and Medicine*.

[88] Eden WV, Thole JER, Zee RVD (1988). Cloning of the mycobacterial epitope recognized by T lymphocytes in adjuvant arthritis. *Nature*.

[89] Moudgil KD, Chang TT, Eradat H (1997). Diversification of T cell responses to carboxy-terminal determinants within the 65-kD heat-shock protein is involved in regulation of autoimmune arthritis. *Journal of Experimental Medicine*.

[90] Shen C-L, Yeh JK, Cao JJ, Tatum OL, Dagda RY, Wang J-S (2010). Synergistic effects of green tea polyphenols and alphacalcidol on chronic inflammation-induced bone loss in female rats. *Osteoporosis International*.

[91] Shen C-L, Yeh JK, Cao JJ, Tatum OL, Dagda RY, Wang J-S (2009). Green tea polyphenols mitigate bone loss of female rats in a chronic inflammation-induced bone loss model. *Journal of Nutritional Biochemistry*.

[92] Rajaiah R, Moudgil KD (2009). Heat-shock proteins can promote as well as regulate autoimmunity. *Autoimmunity Reviews*.

[93] Durai M, Gupta RS, Moudgil KD (2004). The T Cells Specific for the Carboxyl-Terminal Determinants of Self (Rat) Heat-Shock Protein 65 Escape Tolerance Induction and Are Involved in Regulation of Autoimmune Arthritis. *The Journal of Immunology*.

[94] Danieli MG, Markovits D, Gabrielli A (1992). Juvenile rheumatoid arthritis patients manifest immune reactivity to the mycobacterial 65-kDa heat shock protein, to its 180-188 peptide, and to a partially homologous peptide of the proteoglycan link protein. *Clinical Immunology and Immunopathology*.

[95] De Graeff-Meeder ER, Van der Zee R, Rijkers GT (1991). Recognition of human 60 kD heat shock protein by mononuclear cells from patients with juvenile chronic arthritis. *The Lancet*.

[96] Res PCM, Breedveld FC, van Embden JDA (1988). Synovial fluid T cell reactivity against 65 kD heat shock protein of mycobacteria in early chronic arthritis. *The Lancet*.

[97] Miletić T, Kovačević-Jovanović V, Stanojević S (2006). Strain differences and the role for HSP47 and HSP70 in adjuvant arthritis in rats. *Scandinavian The Journal of Immunology*.

[98] Morino M, Tsuzuki T, Ishikawa Y (1997). Specific regulation of HSPs in human tumor cell lines by flavonoids. *In Vivo*.

[99] Chow AM, Brown IR (2007). Induction of heat shock proteins in differentiated human and rodent neurons by celastrol. *Cell Stress and Chaperones*.

[100] Westerheide SD, Bosman JD, Mbadugha BNA (2004). Celastrols as inducers of the heat shock response and cytoprotection. *The Journal of Biological Chemistry*.

[101] Billard C, Izard J-C, Roman V (2002). Comparative antiproliferative and apoptotic effects of resveratrol, *ε*-viniferin and vine-shots derived polyphenols (Vineatrols) on chronic B lymphocytic leukemia cells and normal human lymphocytes. *Leukemia and Lymphoma*.

[102] Lugli E, Ferraresi R, Roat E (2009). Quercetin inhibits lymphocyte activation and proliferation without inducing apoptosis in peripheral mononuclear cells. *Leukemia Research*.

[103] Zunino SJ, Storms DH (2009). Resveratrol alters proliferative responses and apoptosis in human activated B lymphocytes in vitro. *Journal of Nutrition*.

[104] Filippi C, Bresson D, von Herrath M (2005). Antigen-specific induction of regulatory T cells for type 1 diabetes therapy. *International Reviews of Immunology*.

[105] Sakaguchi S, Sakaguchi N, Shimizu J (2001). Immunologic tolerance maintained by CD25+CD4+ regulatory T cells: their common role in controlling autoimmunity, tumor immunity, and transplantation tolerance. *Immunological Reviews*.

[106] Bluestone JA, Abbas AK (2003). Natural versus adaptive regulatory T cells. *Nature Reviews Immunology*.

[107] Shevach EM (2004). Regulatory/suppressor T cells in health and disease. *Arthritis and Rheumatism*.

[108] Frey O, Petrow PK, Gajda M (2005). The role of regulatory T cells in antigen-induced arthritis: aggravation of arthritis after depletion and amelioration after transfer of CD4+CD25+ T cells. *Arthritis Research & Therapy*.

[109] Morgan ME, Sutmuller RPM, Witteveen HJ (2003). CD25+ cell depletion hastens the onset of severe disease in collagen-induced arthritis. *Arthritis and Rheumatism*.

[110] Bettelli E, Carrier Y, Gao W (2006). Reciprocal developmental pathways for the generation of pathogenic effector TH17 and regulatory T cells. *Nature*.

[111] Vojdani A, Erde J (2006). Regulatory T cells, a potent immunoregulatory target for CAM researchers: modulating tumor immunity, autoimmunity and alloreactive immunity (III). *Evidence-Based Complementary and Alternative Medicine*.

[112] Ehrenstein MR, Evans JG, Singh A (2004). Compromised function of regulatory T cells in rheumatoid arthritis and reversal by anti-TNF*α* therapy. *Journal of Experimental Medicine*.

[113] Korganow A-S, Hong J, Mangialaio S (1999). From systemic T cell self-reactivity to organ-specific autoimmune disease via immunoglobulins. *Immunity*.

[114] Cremer MA, Townes AS, Kang AH (1984). Collagen-induced arthritis in rodents. A review of clinical, histological and immunological features. *Ryumachi*.

[115] Ulmansky R, Cohen CJ, Szafer F (2002). Resistance to adjuvant arthritis is due to protective antibodies against heat shock protein surface epitopes and the induction of IL-10 secretion. *The Journal of Immunology*.

[116] Feldmann M, Brennan FM, Foxwell BM, Maini RN (2001). The role of TNF alpha and IL-1 in rheumatoid arthritis. *Current directions in autoimmunity*.

[117] Ivashkiv LB (1996). Cytokine expression and cell activation in inflammatory arthritis. *Advances in Immunology*.

[118] Forsthuber T, Yip HC, Lehmann PV (1996). Induction of TH1 and TH2 immunity in neonatal mice. *Science*.

[119] Stevens DB, Gold DP, Sercarz EE, Moudgil KD (2002). The Wistar Kyoto (RT1^l^) rat is resistant to myelin basic protein-induced experimental autoimmune encephalomyelitis: comparison with the susceptible Lewis (RT1^l^) strain with regard to the MBP-directed CD4+ T cell repertoire and its regulation. *Journal of Neuroimmunology*.

[120] Christen U, Von Herrath MG (2004). Manipulating the type 1 vs type 2 balance in type 1 diabetes. *Immunologic Research*.

[121] Mauri C, Feldmann M, Williams RO (2003). Down-regulation of Th1-mediated pathology in experimental arthritis by stimulation of the Th2 arm of the immune response. *Arthritis and Rheumatism*.

[122] Chun S-C, Jee SY, Lee SG, Park SJ, Lee JR, Kim SC (2007). Anti-inflammatory activity of the methanol extract of Moutan Cortex in LPS-activated Raw264.7 cells. *Evidence-Based Complementary and Alternative Medicine*.

[123] Kim EY, Moudgil KD (2008). Regulation of autoimmune inflammation by pro-inflammatory cytokines. *Immunology Letters*.

[124] Vojdani A, Lambert J The role of Th17 in neuroimmune disorders: target for CAM therapy. Part I.

[125] Ziolkowska M, Koc A, Luszczykiewicz G (2000). High levels of IL-17 in rheumatoid arthritis patients: IL-15 triggers in vitro IL-17 production via cyclosporin A-sensitive mechanism. *The Journal of Immunology*.

[126] Lubberts E, Koenders MI, Oppers-Walgreen B (2004). Treatment with a Neutralizing Anti-Murine Interleukin-17 Antibody after the Onset of Collagen-Induced Arthritis Reduces Joint Inflammation, Cartilage Destruction, and Bone Erosion. *Arthritis and Rheumatism*.

[127] Nakae S, Nambu A, Sudo K, Iwakura Y (2003). Suppression of Immune Induction of Collagen-Induced Arthritis in IL-17-Deficient Mice. *The Journal of Immunology*.

[128] Koenders MI, Kolls JK, Oppers-Walgreen B (2005). Interleukin-17 receptor deficiency results in impaired synovial expression of interleukin-1 and matrix metalloproteinases 3, 9, and 13 and prevents cartilage destruction during chronic reactivated streptococcal cell wall-induced arthritis. *Arthritis and Rheumatism*.

[129] Kasama T, Strieter RM, Lukacs NW, Lincoln PM, Burdick MD, Kunkel SL (1995). Interleukin-10 expression and chemokine regulation during the evolution of murine type II collagen-induced arthritis. *The Journal of Clinical Investigation*.

[130] Halloran MM, Szekanecz Z, Barquin N, Haines GK, Koch AE (1996). Cellular adhesion molecules in rat adjuvant arthritis. *Arthritis and Rheumatism*.

[131] Okamoto H, Kamatani N (2006). A CCR-5 antagonist inhibits the development of adjuvant arthritis in rats. *Rheumatology*.

[132] Barnes DA, Tse J, Kaufhold M (1998). Polyclonal antibody directed against human RANTES ameliorates disease in the Lewis rat adjuvant-induced arthritis model. *The Journal of Clinical Investigation*.

[133] Chen X, Oppenheim JJ, Howard OM (2004). Chemokines and chemokine receptors as novel therapeutic targets in rheumatoid arthritis (RA): inhibitory effects of traditional Chinese medicinal components. *Cellular & Molecular Immunology*.

[134] Nanki T, Shimaoka T, Hayashida K, Taniguchi K, Yonehara S, Miyasaka N (2005). Pathogenic role of the CXCL16-CXCR6 pathway in rheumatoid arthritis. *Arthritis and Rheumatism*.

[135] Cui H-S, Hayasaka S, Zhang X-Y, Hayasaka Y, Chi Z-L, Zheng L-S (2006). Effect of berberine on interleukin 8 and monocyte chemotactic protein 1 expression in a human retinal pigment epithelial cell line. *Ophthalmic Research*.

[136] Jayaprakasam B, Doddaga S, Wang R, Holmes D, Goldfarb J, Li X-M (2009). Licorice flavonoids inhibit eotaxin-1 secretion by human fetal lung fibroblasts in vitro. *Journal of Agricultural and Food Chemistry*.

[137] Lee JS, Park SY, Thapa D, Kim AR, Shin HM, Kim JA HMC05, herbal formula, inhibits TNF-{alpha}-induced inflammatory response in human umbilical vein endothelial cells.

[138] Lee K-H, Yeh M-H, Kao S-T (2009). Xia-Bai-San inhibits lipopolysaccharide-induced activation of intercellular adhesion molecule-1 and nuclear factor-kappa B in human lung cells. *Journal of Ethnopharmacology*.

[139] Mak N-K, Leung C-Y, Wei X-Y (2004). Inhibition of RANTES expression by indirubin in influenza virus-infected human bronchial epithelial cells. *Biochemical Pharmacology*.

[140] Moon MK, Lee YJ, Kim JS, Kang DG, Lee HS (2009). Effect of caffeic acid on tumor necrosis factor-alpha-induced vascular inflammation in human umbilical vein endothelial cells. *Biological and Pharmaceutical Bulletin*.

[141] Yang M-H, Wu F-X, Xie C-M (2009). Expression of CC chemokine ligand 5 in patients with rheumatoid arthritis and its correlation with disease activity and medication. *Chinese Medical Sciences Journal*.

[142] Zaidi SFH, Ahmed K, Yamamoto T (2009). Effect of resveratrol on Helicobacter pylori-induced interleukin-8 secretion, reactive oxygen species generation and morphological changes in human gastric epithelial cells. *Biological and Pharmaceutical Bulletin*.

[143] Chittur S, Parr B, Marcovici G Inhibition of inflammatory gene expression in keratinocytes using a composition containing carnitine, thioctic acid and saw palmetto extract.

[144] Katz S, Harris R, Lau JT-Y, Chau A (2006). The use of gene expression analysis and proteomic databases in the development of a screening system to determine the value of natural medicinal products. *Evidence-Based Complementary and Alternative Medicine*.

[145] Matsumoto C, Kojima T, Ogawa K (2008). A proteomic approach for the diagnosis of ’Oketsu’ (blood stasis), a pathophysiologic concept of Japanese traditional (Kampo) medicine. *Evidence-Based Complementary and Alternative Medicine*.

